# Dendrimer-Mediated Delivery of DNA and RNA Vaccines

**DOI:** 10.3390/pharmaceutics15041106

**Published:** 2023-03-30

**Authors:** Lyubov A. Kisakova, Evgeny K. Apartsin, Lily F. Nizolenko, Larisa I. Karpenko

**Affiliations:** 1 State Research Center of Virology and Biotechnology VECTOR, Rospotrebnadzor, 630559 Kol’tsovo, Russia; orlova.lyub1996@yandex.ru (L.A.K.); nizolenko@inbox.ru (L.F.N.); 2 CBMN, UMR 5248, CNRS, Bordeaux INP, University Bordeaux, F-33600 Pessac, France

**Keywords:** dendrimer, DNA vaccine, mRNA vaccine, vaccine delivery, clinical trials

## Abstract

DNA and RNA vaccines (nucleic acid-based vaccines) are a promising platform for vaccine development. The first mRNA vaccines (Moderna and Pfizer/BioNTech) were approved in 2020, and a DNA vaccine (Zydus Cadila, India), in 2021. They display unique benefits in the current COVID-19 pandemic. Nucleic acid-based vaccines have a number of advantages, such as safety, efficacy, and low cost. They are potentially faster to develop, cheaper to produce, and easier to store and transport. A crucial step in the technology of DNA or RNA vaccines is choosing an efficient delivery method. Nucleic acid delivery using liposomes is the most popular approach today, but this method has certain disadvantages. Therefore, studies are actively underway to develop various alternative delivery methods, among which synthetic cationic polymers such as dendrimers are very attractive. Dendrimers are three-dimensional nanostructures with a high degree of molecular homogeneity, adjustable size, multivalence, high surface functionality, and high aqueous solubility. The biosafety of some dendrimers has been evaluated in several clinical trials presented in this review. Due to these important and attractive properties, dendrimers are already being used to deliver a number of drugs and are being explored as promising carriers for nucleic acid-based vaccines. This review summarizes the literature data on the development of dendrimer-based delivery systems for DNA and mRNA vaccines.

## 1. Introduction

Vaccines are among the most effective means of achieving the epidemic well-being of the population. A good vaccine platform should ensure the formation of effective long-term immunity, be simple and fast to develop, reproducible, thermostable, and relatively inexpensive to manufacture and use, but should not cause un-desirable side reactions. Nucleic acid-based vaccines (mRNA and DNA vaccines) meet many of these requirements; they are promising tools compared to traditional vaccination platforms due to their unique properties [1,2].

The COVID-19 pandemic has stimulated the development of these platforms. The first mRNA-based vaccines against infectious diseases, developed by Moderna and Pfizer/BioNTech, have been approved for human vaccination against COVID-19 in 2020 [3,4]. The DNA vaccine against COVID-19, developed by Zydus Cadila, became the first DNA vaccine in the world to be approved for human vaccination in 2021 [5,6].

Among the advantages of DNA vaccines, it should be noted that, similar to vector and attenuated vaccines, they effectively induce T-cell immunity, while having a fairly high safety profile compared to that of vector vaccines [7,8,9]. The cost-effective production of DNA vaccines, the possibility of their rapid adaptation to new targets, and stability at ambient temperature, which favorably distinguishes them from mRNA vaccines that require storage at low temperatures, are also worth mentioning [7,10]. 

Messenger RNA vaccines have a number of attractive features, including the simplicity of design, low production cost, and low reactogenicity [11]. The mRNA lacks the immunogenic CpG motifs that are present in DNA vaccines. They do not need to cross the nuclear membrane to allow expression of the target protein, since they are delivered to the cytoplasm. The inability of mRNA to be integrated into the host genome rules out insertional mutagenesis, making delivery safer than for plasmid DNA [1,12,13,14]. 

A certain disadvantage of both DNA and mRNA vaccines is their low immunogenicity when administered as naked plasmid DNA or mRNA [7,8,9]. To exhibit specific activity, mRNA must penetrate into the cytoplasm of the target cell, and the DNA vaccine must penetrate into the cell nucleus. However, cell membrane features in eukaryotic cells prevent spontaneous penetration of NAs directly into cytoplasm. Furthermore, free NAs, especially mRNAs, are rapidly digested by endogenous nucleases in biological fluids and tissues. These factors call for technological approaches for specific DNA/RNA delivery into immune cells. 

A wide range of strategies have been tried for the delivery of DNA vaccines, including viral carriers, liposomal packaging, the use of adjuvant plasmids encoding cytokines, and delivery by gene gun, electroporation, or injection. These methods help to solve the problem of immunogenicity, but are sometimes associated with some safety problems, technological difficulties, and an increase in the cost of the developed vaccine [5,15,16,17,18].

Various forms of polycationic carriers are used for mRNA delivery, including lipids, polymeric and polypeptide systems, dendrimers, gold nanoparticles, and hybrid systems [19,20,21]. Lipid nanoparticles are currently one of the most commonly used mRNA delivery vehicles. Almost all developers of mRNA vaccines against SARS-CoV-2, including Moderna/NIH and Pfizer/BioNTech, encapsulate RNA molecules in lipid nanoparticles [22]. The main problem associated with the delivery of mRNA by liposomes is related to the nature of lipids. In particular, positively charged liposomal particles can bind to negatively charged proteins and nucleic acids and attach to the cell surface, thereby contributing to the destabilization of the plasma membrane and causing side effects in a vaccinated person [23,24]. Another problem of lipid nanoparticles is their sensitivity to freezing and thawing during manipulation, which makes it difficult to use them for mass vaccination [25,26].

Thus, the problem of delivering vaccines based on nucleic acids has not yet been finally resolved. Therefore, research is being actively conducted to develop various alternative methods for the delivery of DNA and RNA vaccine constructions. A promising direction is the use of cationic dendrimers, hyperbranched three-dimensional macro-molecules with perfectly defined molecular structure. Herein, we review recent progress in the use of dendrimers for the delivery of DNA and mRNA vaccines against various diseases. In view of the potential use of dendrimers in humans as components of vaccine formulations, we pay special attention to the species currently subjected to clinical trials or approved for use in humans. Validation of biosafety and biological activity of dendrimers in clinical trials will be also discussed. 

## 2. The Use of Dendrimers for Biomedical Applications

### 2.1. Dendrimer Structure and Properties

**Figure 1:**
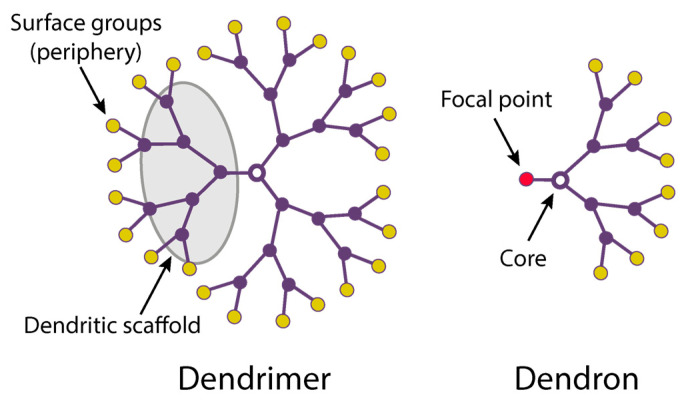
Schematic drawings of a dendrimer and dendron.

Dendrimers are hyperbranched macromolecules composed of monomers radially emanating from a central core [27] (Figure 1). Due to their repetitive structure, dendrimers can be considered polymers, but in contrast to polymers, they are never synthesized by polymerization reactions, but step-by-step in an iterative fashion, creating layers. Each layer is called “generation” [28,29]. Higher-generation dendrimers (G > 3) have a near-globular shape [30].

Owing to their peculiar synthesis, the structure of dendrimers is perfectly defined and highly reproducible. An isolated branch of a dendrimer is called a “dendron”. By choosing structural elements of dendrimers, one can opt for their physicochemical properties, adjust the molecular weight, functional group density, as well as modify dendrimers with different functionalities. The synthetic flexibility allows one to optimize their structure according to a specific task.

Dendrimers are a promising platform for designing functional species owing to their multivalency, which is reminiscent of the multivalent interactions widely found in nature. Multivalent interactions can be collectively much stronger than the corresponding monovalent interactions, in particular through multiple ligand–receptor interactions. Dendrimers, which are inherently multivalent species, are widely used in different fields of science and technology, such as catalysis [31], biomaterials [32,33], regenerative and cell biology [34,35,36,37], and nanomedicine [38,39,40,41].

One of the most intriguing properties of dendrimers and dendrons is the so-called “dendritic effect” [42], i.e., a dramatic increase in efficiency when using a dendrimer compared to monomer. The dendritic effect is influenced by the nature and size of the core as well as by the cooperativity of functional groups at the periphery. The dendritic effect-enhanced multivalency and structure uniformity are the major differences between dendrimers and hyperbranched polymers. They frequently define the dendrimer properties and behavior in different processes at the nanoscale level.

Apart from dendrimers, the use of dendrons is considered highly promising. Unlike dendrimers, dendrons have two topologically and chemically different sites: the focal point and the periphery, which enables their selective functionalization with various moieties, depending on the desired application. Such a feature allows dendrons to exhibit enhanced functionality compared to that of dendrimers. Today, dendrons have been applied as agents for imaging, catalysis [43,44,45], healthcare [46,47,48,49,50], nanoparticle stabilization and functionalization [51,52,53,54,55], surface modification [56,57], etc.

Amphiphilic dendritic species are an emerging class of dendrons holding great potential in a wide range of applications. By varying the nature and the structure of a functional moiety in the focal point, one can achieve the controllable self-assembly of dendrons into supramolecular associates [55,58,59,60]. Such molecules, which are in fact Janus-type particles, expand the functionality of dendrimers for various applications. For instance, amphiphilic dendrons have been shown to deliver chemotherapy drugs [61,62,63] and anti-cancer siRNAs [64,65,66] in tumors, thus efficiently suppressing tumor growth. Controllable assembly of complex supramolecular structures mimicking the cell surface [67], along with the possibility to decorate them with functional moieties, nanoparticles, or (bio)macromolecules [68] suggests that amphiphilic dendrons are highly emerging for designing precisely engineered biomaterials.

Most dendrimers currently in use for biomedical applications are composed of organic branches and generally mimic biopolymers: poly(amidoamine) dendrimers (PAMAM), poly(propylene imine) dendrimers (PPI), and poly(L-lysine) dendrimers (PLL) etc. [29,69,70,71,72,73,74,75,76,77,78,79]. Furthermore, there are dendrimers containing main group heteroatoms as core components and branching points [31], such as silicon [80] or phosphorus [81], as well as their combinations with organic branches (PAMAM-organosilicon dendrimers (PAMAMOS)), phosphorus-viologen dendrimers [82], etc.

The major advantage of dendrimers and dendrons compared to other types of synthetic polymers is the precisely defined molecular structure [83], which facilitates their certification for use in drug formulations and medical devices. Furthermore, dendrimers and dendrimer-based nanoformulations are efficiently taken up by different cell types through clathrin-mediated endocytosis pathway [84]. In order to improve biocompatibility and biological activity, as well as achieve active targeting, dendrimers can be functionalized by various chemical and biological entities, such as polyethylene glycol, folate, signal peptides, ligands of cell surface receptors, etc. [83]. Reporter groups (fluorescent dyes, radioactive, or MRI-active groups) are introduced to monitor the biodistribution of dendrimer-based formulations. Rich dendrimer chemistry makes it possible to flexibly choose conjugation methods. The presence of multiple functional groups on the dendrimer surface gives room for obtaining multifunctional agents bearing different types of functional groups in a single construct. This helps decrease side effects of formulations [85].

Bioactive molecules can be either conjugated or complexed to dendrimers, and the complexation may involve hydrophobic or electrostatic interactions. In the case of nucleic acid therapeutics, they are usually bound by electrostatic interactions to dendrimers bearing cationic groups on the surface to form polyelectrolyte complexes [86].

### 2.2. Validation of Dendrimers in Clinical Trials

Distinct dendrimer-based constructions have been subjected to clinical trials; their safety and efficacy have been demonstrated in Phases 1–3, although some studies are still ongoing. Table 1 lists the dendrimer-based products involved in clinical trials, according to the clinicaltrials.gov database. These examples clearly highlight the growing employment of dendrimer-based drug delivery systems in the pharmaceutical industry.

**Figure 2:**
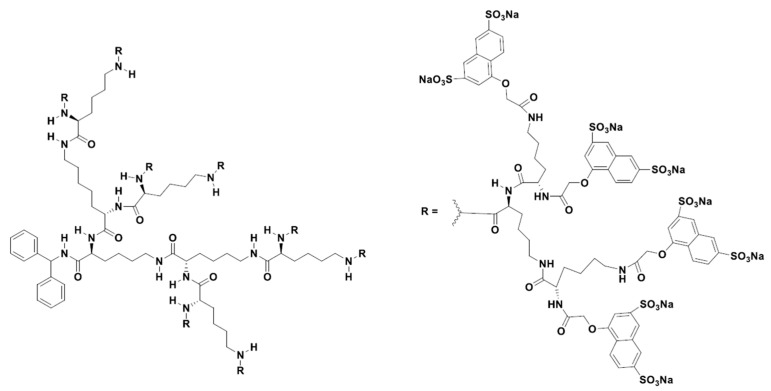
Two-dimensional chemical structure of VivaGel^®^ [118].

The first dendrimer-based, anti-microbial drug approved for use in humans was Vivagel^®^ (SPL7013) (Starpharma, Abbotsford, Australia), a poly-L-lysine dendrimer derivastive used as a topical microbicide for the prevention and treatment of bacterial vaginosis (BV). There have been at least 13 clinical studies of this product, with the first clinical trial completed in 2004. SPL7013 is a polyanionic dendrimer with a benzylhydramine lysine core and has 32 naphthalene disulfonate surface functionalities (Figure 2). This product also provides the prevention of genital herpes (HSV-2), HIV, and other sexually transmitted infections (STIs). Studies have shown that SPL7013 is generally well tolerated with minimal side effects [108,109,110,111,112,113,114,115,116,117]. SPL7013, in the form of a nasal spray called VIRALEZE, has also undergone a first growth phase as registered in the Australian and New Zealand Clinical Trials Registry [118].

Another example of a poly-L-lysine dendrimer developed by Starpharma and AstraZeneca is AZD0466. This is a fifth generation poly-L-lysine dendrimer, a highly optimized molecule containing the anti-cancer drug AZD4320, and a PEGylated poly-L-lysine dendrimer. AZD0466 belongs to a new class of oncology drugs that provide efficient delivery of a dual Bcl-2/xL inhibitor with an optimized release profile that is designed to reduce the potential for toxicity associated with dual Bcl-2/xL inhibition [119,120,121,122]. The AZD0466 construct is currently undergoing simultaneous phase1/phase 2 clinical trials in patients with advanced hematologic malignancies as monotherapy or in combination with certain combination therapies, such as antifungals.

Another example is a fifth-generation poly-L-lysine-based dendrimer named ImDendrim, which has proven to be suitable for encapsulating rhenium and radioactive 188Re. This potential radiopharmaceutical agent showed antitumor activity in liver cancer in mice [123]. The clinical study of this drug concerns patients with an inoperable liver tumor that is resistant to other classical chemotherapy.

PAMAM dendrimers [70,124,125,126,127,128,129] are the most widely used dendrimers. In their native form, they can be easily modified as desired. However, low storage stability has long precluded the use of PAMAM dendrimers in clinical trials [130]. However, PAMAM dendrimers have recently passed clinical trials proposed by Ashvattha Therapeutics.

The first PAMAM dendrimer-based injectable is OP-101, a covalent conjugate of generation 4 hydroxyl-terminated poly(amidoamine) dendrimer and N-acetylcysteine (NAC) developed by the American firm Ashvattha Therapeutics and its subsidiary Orpheris, Inc. Safety trials for intravenous and subcutaneous injections in healthy volunteers began in 2018 and continued into 2020. The study is not yet complete, but preliminary results indicate that OP-101 is well tolerated and may have the potential to treat systemic inflammation and neuronal damage, reducing the disease severity and mortality in patients [131].

Another drug from the same company, Ashvattha Therapeutics, with the open name D-4517.2, is a hydroxyl dendrimer, a VEGFR tyrosine kinase inhibitor. Initial studies have shown that this drug is safe and well tolerated, with only a few patients experiencing mild transient reactions at the injection site associated with the technique [132].

**Figure 3:**
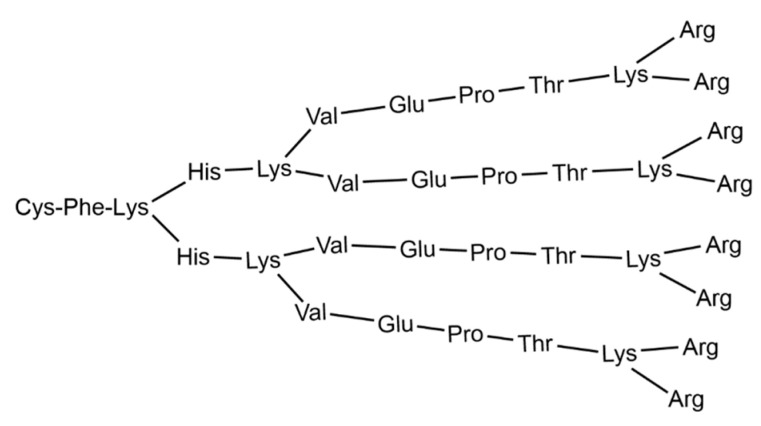
Structure of the cationic peptide dendrimer KK-46, usable as carrier of siCoV [133].

Two clinical trials against COVID-19 were conducted in 2021, both based on the same cationic peptide dendritic structure containing lysine as branch elements, named KK-46, the structure of which is shown in Figure 3.

This compound, developed by the Institute of Immunology of the Federal Medical and Biological Agency of Russia, in collaboration with the St. Petersburg Research Institute of Vaccines and Serums, is used as a carrier of siRNA, modified to suppress SARS-CoV-2 (siCoV) by inhibiting its replication [134]. The association of KK-46 with siCoV has been named MIR 19^®^ [135]. Based on preclinical data, the researchers hypothesized that SARS-CoV-2 inhibition by siCoV/KK46 could potentially reduce lung inflammation, thereby improving treatment outcomes. According to the test results, the drug was registered and introduced into civil circulation by the Ministry of Health of the Russian Federation on 22 December 2021 LP-007720 as a direct-acting antiviral agent [136].

## 3. Delivery of DNA and mRNA Vaccines Using Dendrimers

To date, a number of dendrimer-containing candidate DNA or mRNA vaccine formulations against viral, bacterial, and parasitic infections, as well as oncological diseases, have been reported (Table 2). Below, we discuss in detail the development of distinct types of vaccine formulations: from physicochemical properties to in vivo studies. 

### 3.1. Dendrimers for the Delivery of DNA Vaccines against Viral Infections

**Figure 4:**
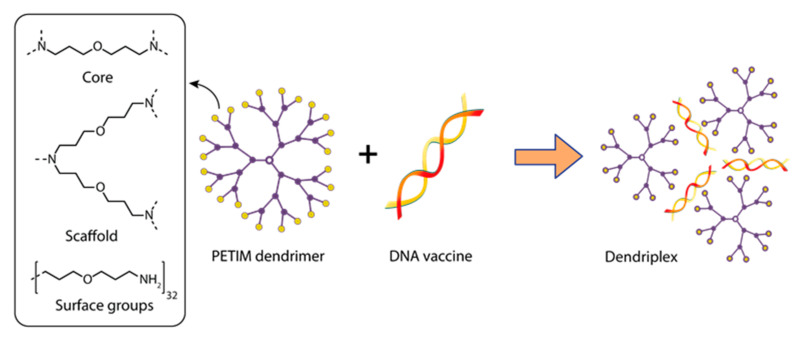
Constructing vaccine nanoformulations based on PETIM dendrimers.

Ullas et al. [137] focused on developing candidate vaccines against the rabies virus. The authors used the polyetherimine dendrimer (PETIM) to increase the immunogenicity of the pIRES-Rgp DNA vaccine, encoding the full-length rabies virus glycoprotein gene in order to increase its immunogenicity. PETIM is a fourth-generation amino-terminated dendrimer, a globular nanopolymer with an approximate diameter of 3.5 nm, and a low cytotoxicity profile (Figure 4).

The DNA vaccine pIRES-Rgp, in a complex with dendrimers, showed significantly higher immunogenicity when mice were immunized with either the naked plasmid pIRES-Rgp or the PETIM-pIRES-Rgp complex [137]. Rabies virus neutralizing antibody (RVNA) titers in immune sera were assessed using the rapid fluorescence focus inhibition test. Protective levels of RVNA titer (≥0.5 IU/mL) were observed by day 14 similar to in the animals immunized with naked pIRES-Rgp and its complex with dendriplex. However, immunization with the PETIM-pIRES-Rgp dendriplex induced a 4.5-fold higher RVNA titer compared to pIRES-Rgp, while the antibodies had virus-neutralizing activity. It is likely that the dendriplex size (~500 nm) facilitated its efficient uptake by antigen-presenting cells near the site of inoculation, which led to efficient release of the antigen and development of antibody responses. Sustained high levels of RVNA titers on day 90 in the groups immunized with the PETIM-pIRES-Rgp dendriplex provide additional evidence for the possibility of antigen retention and slow antigen release by the encapsulated nanospheres. Importantly, the protective efficacy provided by the PETIM-pIRES-Rgp complex was comparable to that of the inactivated virus vaccine (Rabipur). The authors believe that the PETIM-pIRES-Rgp vaccine will be easier and cheaper to manufacture, more stable and resistant to higher ambient temperatures during storage, and thus more suitable for resource-limited settings than the commercial Rabipur vaccine [137].

**Figure 5:**
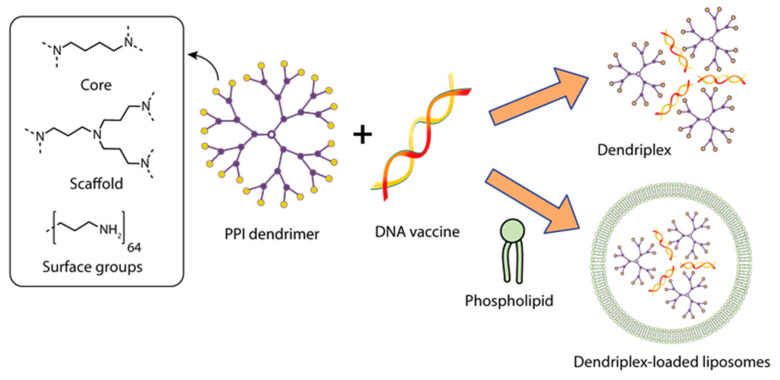
Constructing vaccine nanoformulations based on PPI dendrimers.

Dutta et al. [138] investigated the potential of dendrimers and dendrosomes in genetic immunization against hepatitis B using the pRc/CMV-HBs[S] DNA vaccine encoding the hepatitis B surface antigen (HBsAg) sequence (5.6 kb). Several complexes of pRc/CMV-HBs plasmid with G5 poly(propylene imine) dendrimer (PPI) were obtained, differing in the plasmid: dendrimer ratio (Figure 5).

The complex with a DNA: dendrimer ratio of 1:50 (PPI 50), which was subsequently chosen to obtain dendrosomes, had the highest statistically significant transfection efficiency in vitro. To obtain dendrosomes, phosphatidylcholine (PC) and cholesterol (C) were dissolved in diethyl ether at various molar ratios; a solution of the optimized dendrimer-DNA complex in PBS was added, sonicated, and homogenized to obtain particles sized ~200 nm. The authors showed that the optimal capture efficiency (the ratio between the amount of captured PPI-DNA complex and the amount of lipid complex added, expressed as a percentage) of 46.79 ± 1.33% was achieved at a 7:3 molar ratio of phosphatidylcholine to cholesterol (PC:C) in dendrosome composition DF3. DF3 was shown to have the optimal vesicle size, zeta potential, and uptake efficiency during the transfection of CHO cells, which was determined by the production of HBsAg [138].

The immunogenicity of the vaccine was studied using Balb/c mice as a model. The first group of animals received a single intramuscular injection of 10 μg of the naked plasmid pRc/CMV-HBs (DNA vaccines); the second group of animals was immunized with the PPI 50 dendrimer complex; and the third group was immunized with the DF3 dendrosome. The immunogenicity test was based on anti-HBsAg antibody titer assessment. It was shown that the DF3 complex elicited the maximum immune response of both total IgG and its studied subclasses (IgG1, IgG2a, and IgG2b) compared to naked DNA vaccine and PPI 50. The lowest antibody titer was observed in the animals immunized with naked plasmid pRc/CMV-HBs. In addition, the animals immunized with pRc/CMV-HBs and PPI 50 showed an abrupt decrease in antibody titer after six weeks, while the DF3 group had stable levels of IgG1, IgG2a, and IgG2b after six weeks, thus proving its effectiveness in maintaining antibody titer and protection against hepatitis B [138].

The animals immunized with the dendrimer and dendrosome formulations exhibited a Th1 immune response, further evidenced by higher IgG2a/IgG1 ratios. Cellular immune response was measured according to the content of endogenous IFN-γ in the spleen of mice immunized with pRc/CMV-HBs, PPI 50, and DF3, which were injected intravenously with 1 mg of the encoded antigen 24 hours before harvesting. The animals immunized with PPI 50 and DF3 complexes produced a significantly higher level of IFN-γ, which indicates the formation of a Th1-cell response [138].

**Figure 6:**
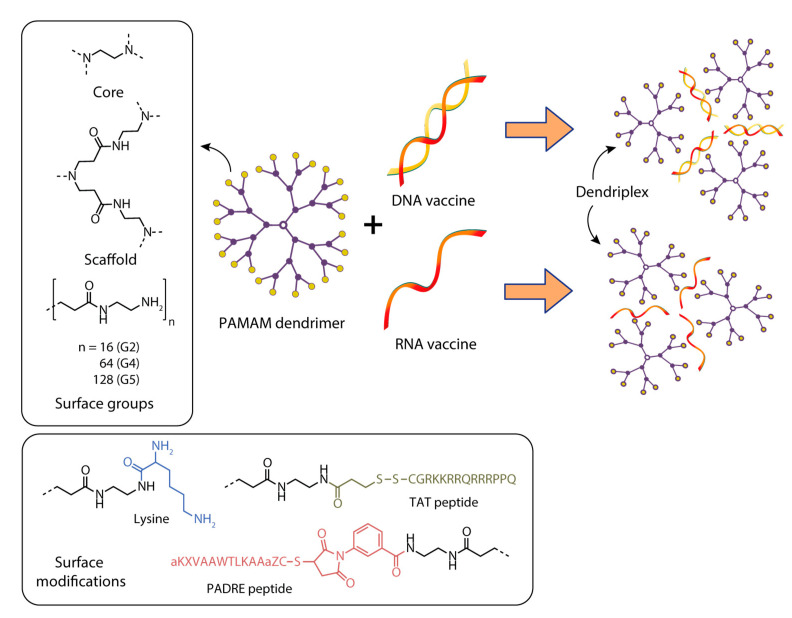
Constructing vaccine nanoformulations based on PAMAM dendrimers.

Karpenko et al. [139] studied the delivery of a DNA vaccine against the Ebola virus using the fourth-generation polyamidoamine dendrimers (PAMAM G4) and a polyglucin:spermidine (PG) conjugate (Figure 6).

DNA vaccines are present among plasmids encoding artificial multi-epitope T-cell antigens EV.CTL and EV.Th, consisting of conserved epitopes of the EBOV GP, VP24, VP30, VP35, VP40, NP, and L Ebola virus proteins [139].

The physicochemical properties of complexes of polycationic polymers with DNA vaccines at different plasmid:polymer ratios were studied. Taking into account both the completeness of DNA binding and the size of the resulting complexes, the following ratio was chosen for biological tests: 3:1 for PAMAM. Since the DNA was taken in a charge excess, the surface charge of the resulting constructs remained negative after complex formation. The resulting complexes are nanosized structures with sizes of 100–300 nm for PG and <100 nm for PAMAM [139].

The study focusing on immunogenicity of the resulting constructs showed no statistically significant differences in the ability to elicit virus-specific T-cell responses between the groups of mice immunized with naked DNA vaccines and in combination with PAMAM G4. At the 3:1 ratio, PAMAM used as a packaging agent did not significantly improve the immunogenicity of the DNA vaccine. The authors believe that the complex formation of PAMAM with plasmids needs further optimization [139].

The use of native dendrimers and their conjugates with various proteins for the delivery of DNA vaccines is currently being studied.

Bahadoran et al. [140] studied the efficiency of delivery of a DNA vaccine against the H5N1 influenza virus (pBud-H5-GFP-IRF3) as part of a complex with PAMAM polyamidoamine dendrimer or a dendrimer conjugated with the TAT-PAMAM transcription transactivator TAT (Figure 6).

The TAT–PAMAM polyplexes showed approximately 10 mV higher zeta potential values than the PAMAM polyplexes. A comparison of the transfection efficiencies of eukaryotic cells in vitro revealed that the pDNA–TAT–PAMAM complex is more efficient and less toxic than pDNA–PAMAM. The authors suggest that TAT-conjugated dendrimers have a higher charge density and, therefore, can form positively charged polyplexes, which are considered important for their adsorption on negatively charged cell membranes followed by an uptake by cells through internalization mechanisms [140].

To evaluate the immune response, mice were immunized with pBud-H5-GFP, PAMAM/pBud-H5-GFP, TAT-PAMAM/pBud-H5-GFP, and TAT-PAMAM/pBud-H5-GFP-IRF3 constructs. The expression levels of the H5 gene in the blood were determined on days 3 and 7 after immunization. Significantly higher expression of the H5 gene was detected in the group of animals immunized with the DNA vaccine in combination with the dendrimer-TAT conjugate. In the same group of mice, a higher titer of specific antibodies was observed compared to the level of antibodies in animals immunized with pDNA–PAMAM [141].

Similar results were obtained in the study of cellular response, which was assessed using flow cytometry. The largest cellular response was induced by the TAT-PAMAM/pBud-H5-GFP-IRF3 construct.

The use of the TAT peptide resulted in more than a doubling of the number of CD8^+^ T lymphocytes. The effect of the TAT peptide on CD4^+^ T cells was not as significant as on CD8^+^ T cells [141]. 

Thus, taking into account such properties as high transfection efficiency with relatively low cytotoxicity and ease of preparation, the TAT-PAMAM dendrimer is a promising non-viral vector together with IRF3 as a genetic adjuvant to induce appropriate immune responses. Further research is needed to determine whether improved immune responses can protect mice after a lethal dose of the H5N1 virus [141].

### 3.2. Dendrimers for mRNA Delivery of Vaccines against Viral Infections

There is a very limited number of studies on mRNA vaccines against viral diseases using dendrimers. 

**Figure 7:**
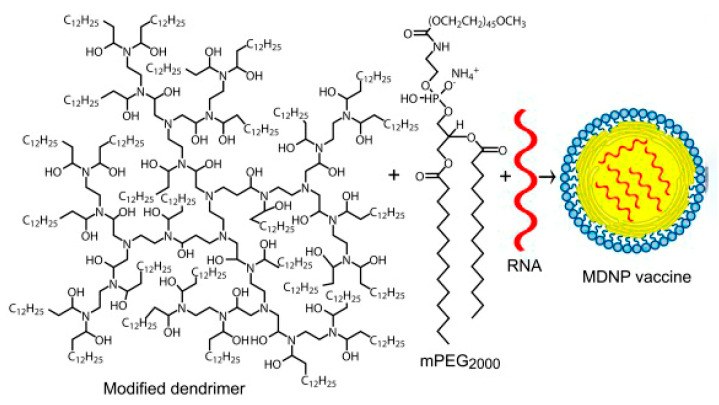
The structure of the amphiphilic dendrimer used for preparing MDNP [142].

Chahal et al. [142] reported the results of studies on the use of a modified PAMAM dendrimer bearing multiple aliphatic chains for the delivery of mRNA replicons against three viral infections (Ebola virus, H1N1 influenza, and Toxoplasma gondii) and their delivery using a modified dendrimer molecule (Figure 7) as follows: an ionizable dendrimer-based nanomaterial, lipid-anchored PEG, and RNA are combined to form the final vaccine-modified dendrimer nanoparticle (MDNP). The vaccine constructs were self-replicating RNAs derived from Venezuelan equine encephalitis virus (VEEV) as a vector, with sequences encoding the Ebola virus, H1N1 influenza, or Toxoplasma gondii antigens inserted into its genome. The resulting mRNA replicons were encapsulated in MDNP, and their activities were examined both in vitro and in vivo. 

MDNP-encapsulated RNAs have been successfully expressed in a wide range of cell culture types, including the HeLa human epithelial cell line, mouse and human primary fibroblasts, a mouse dendritic cell line, etc.

C57BL mice were immunized with MDNP VEEV with the Ebola virus glycoprotein gene. Animals were challenged with a lethal dose of mouse-adapted EBOV (ma-EBOV) 28 days after immunization. All control animals succumbed to EBOV infection by day 7, with 100% survival achieved by a single immunization with 40 μg MDNP, and no clinical pathological findings of EBOV were observed during the study. Protection was only attenuated at doses of MDNP vaccine below 40 µg, but 60% of the animals survived after immunization with 40 µg MDNP. Comparable humoral and protective reactions were caused by the introduction of a “naked” replicon, that is, a 100-fold increase in the total amount of RNA [142].

A similar experiment was carried out for influenza. BALB/c mice were immunized with MDNP VEEV encoding influenza H1N1 hemagglutinin (A/WSN/33) and challenged with a lethal dose of virus 14 days later. Control mice died from the infection by day 7, while animals immunized with the nanoparticles survived the challenge and showed complete recovery of body weight by day 11.

A multiplex vaccine against *Toxoplasma gondii* was created using the same principle. No toxoplasmosis vaccine currently exists, so it is impossible to provide absolute prevention [150].

Six self-replicating VEEV mRNAs, each encoding one parasite antigen (GRA6, ROP2A, ROP18, SAG1, SAG2A, and AMA1), were pooled at equal molar ratios using a monodisperse ionizable dendrimer nanoparticle. Selected antigens appear at several stages of the life cycle of the parasite and are common to several strains. BALB/c mice were immunized intramuscularly with a single 40 μg vaccine dose and exposed to lethal doses of the parasite 30 days post-immunization. The animals were followed up for clinical symptoms of the disease. On day 12, all the immunized mice survived the infection. Using the model of transgenic mice OT-1/Rag1−/−, the authors showed that this system is capable of inducing responses not only of antibodies, but also of CD8^+^ T cells. It is also interesting that such a vaccine does not require an adjuvant and is administered once [142].

Thus, a fully synthetic single-dose delivery platform has been created; it requires adjuvants and allows the use of vaccine constructs with multiple replicons expressing different antigens. After a single immunization, these contaminant-free, rapid-production vaccines elicit vital CD8^+^ T cell and antibody responses that provide complete protection against exposure to even lethal pathogens. According to the authors, this technology may allow one to create rapid-response vaccines with broad efficacy, which will reduce the number and frequency of vaccinations [142].

The dendrimer vaccine technology developed by the same group of researchers was used to create a candidate RNA vaccine against the Zika virus [143]. The vector replicon RNA encoding the premembrane (prM) and envelope (E) proteins of the ZIKV Z1106033 isolate as a single open reading frame was compiled and packaged using the MDNP technology. Mice were immunized on day 0 and boosted 5 weeks later with doses of 40 μg (based on RNA weight) of the nanoparticle vaccine by intramuscular injection. Immunization induced the formation of a high titer of specific IgG to the ZIKV protein and CD8^+^ cells, specifically recognizing the conserved T-cell epitope of IGVSNRDFV. The authors emphasize that their approach can be used to evaluate new candidate antigens and identify immune correlates without using the live Zika virus [143].

### 3.3. Dendrimers for the Delivery of DNA Vaccines against Bacterial Infection

A number of publications have focused on the delivery of DNA vaccines encoding antigens of various bacterial infectious agents.

**Figure 8:**
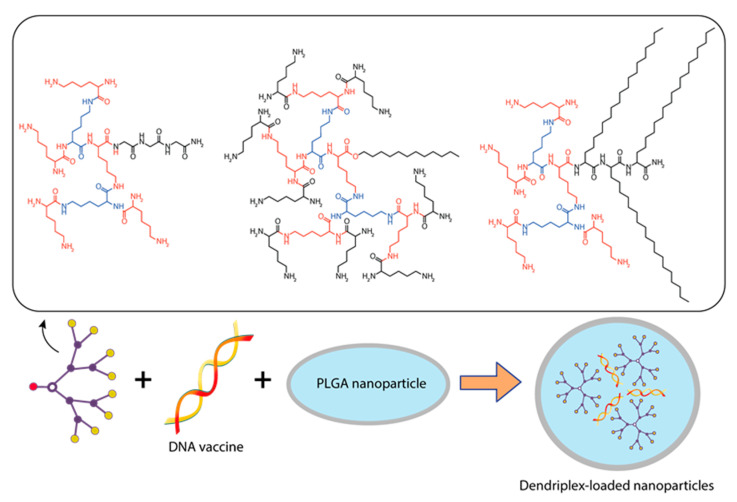
Constructing vaccine nanoformulations based on glycine-polylysine dendrons and their amphiphilic variants.

Ribeiro et al. [144] encapsulated dendriplexes containing a dendrimer:plasmid complex encoding the *Bacillus anthracis* protective antigen (PA) gene into poly(lactide-co-glycolide) (PLGA) particles using the double emulsion method. They used two types of dendrons: a dendron with three C_18_ chains (C_18_ dendron) and a dendron without any hydrocarbon chains attached (C_0_ dendron) (Figure 8).

Three types of particles were investigated, namely PLGA-C18 dendriplexes, PLGA-C0 dendriplexes, and a pDNA control system without PLGA. Dendron:pDNA complexes were encapsulated in PLGA particles at a ratio of 10:1 to assess antibody production, primarily because they have the smallest diameter at low dendron concentrations, which also minimizes their toxicity [144].

Research into the immunogenicity of dendriplexes showed that the antibody response to both PLGA PA-C_18_ and PLGA PA-C_0_ particles gradually increased over a 9-week period. It was likely to occur due to the booster effect in addition to the delayed release of DNA from the PLGA particles and, therefore, transfection of muscle cells to promote antigen presentation [144].

Mice that had received PLGA particles containing naked DNA-PA were only able to generate a weak response against PA. Animals in both groups that were immunized with PLGA PA-C_0_ and PLGA PA-C_18_ had higher anti-PA IgG titers than those immunized with PLGA-PA. The highest level of IgG antibodies against PA was observed in the serum of animals in the PLGA-C_18_ group. However, none of the sera of animals immunized with PA-C_18_, PA-C_0_, or PA-PLGA were able to neutralize the toxin. Thus, the mice lacked protection against a lethal dose of the toxin and infection with *Bacillus anthracis.* The authors believe that further studies are needed to optimize the composition of DNA vaccines and increase the level of antibodies against the lethal toxin as well as their functionality [144].

Verminnen et al. [145] focused on developing an aerosolized DNA vaccine to protect turkeys against *Chlamydophila psittaci* infection. pcDNA1/MOMP_opt_–EGFP plasmids were constructed to test the efficiency of transfection, and pcDNA1/MOMP_opt_ containing optimized sequences of the transgene and regulatory elements. For packaging the DNA vaccine, the authors used plasmid complexes with various cationic polymers, such as G2 and G5 PAMAM dendrimers (Figure 4), linear and branched polyethyleneimine (lPEI and brPEI), and lipoplexes with DOTAP/DOPE cationic liposomes [145].

An evaluation of the efficiency of in vitro transfection with the plasmid pcDNA1/MOMP_opt_–EGFP, in combination with different polymers, showed that, although the plasmid:lPEI complex had the best results, it was completely destroyed in the nebulizer, and PAMAM did not increase the transfection ability of the plasmid. The efficiency of lipoplexes were also statistically unreliable. Therefore, a plasmid complex with a branched polyethyleneimine (brPEI) resembling dendrimers but lacking their regular structure was chosen for further work [145].

DNA vaccine immunogenicity testing was performed in SPF turkey models that had been vaccinated either intramuscularly (IM) or nebulised, followed by challenge with 10^8^ TCID_50_ of *Cp. psittaci* avian genotype D. Turkeys were divided into three groups: groups one and two were immunized intramuscularly with naked pcDNA/MOMP_opt_ or brPEI-pcDNA/MOMP_opt_ complex, respectively, and group three was immunized aerogenically by spraying brPEI pcDNA/MOMP_opt_ through a nebulizer [145].

For the IM administration of the brPEI-pcDNA1/MOMP_opt_ complexes, the immune response was higher than for the case when these complexes were introduced by aerosol (the average titers of MOMP-specific total serum antibodies per group were 85.0 ± 83.6 and 45.0 ± 17.3). However, the immunogenicity of the aerosolized complex was higher than that of the naked pcDNA1/MOMP_opt_ injected IM (30.0 ± 24.5). Meanwhile, a significant level of protection against infection was observed in all the immunized turkeys. Severe clinical signs and lesions occurred only in intact control animals. However, turkeys immunized with brPEI-pcDNA1/MOMP_opt_ IM appeared to be more protected than turkeys aerosolized with this complex. The authors hypothesized that the birds simply inhaled only a fraction of the 500 micrograms administered per bird. Accordingly, the method of delivering the vaccine construct must be adapted to obtain a more homogeneous distribution of the vaccine in the upper and lower respiratory tract of birds, and to reduce the vaccine dose. At the same time, there were no statistically significant differences in tissue lesions, the presence of chlamydial antigen, or isolation of chlamydia in turkeys immunized intramuscularly with naked pcDNA1/MOMP_opt_ (group 1) and turkeys immunized aerogenically with brPEI-pcDNA1/MOMP_opt_ (group 3). Protection correlated with a high B-cell response upon immunization, with an “early” secondary serum antibody response upon challenge, and with a high proliferative response, especially of CD4^+^ T cells [145].

### 3.4. Dendrimers for the Delivery of DNA Vaccines against Parasitic Infections

Creating anti-parasitic vaccines is not an easy task. It is no coincidence that a number of researchers have high hopes for new types of vaccines, such as DNA vaccines. Of course, the question inevitably continues to arise here about finding ways to effectively deliver them.

The aim of the research by Wang et al. [146] was to evaluate the effectiveness of lysine-modified PAMAM for delivering DNA vaccines against Japanese schistosomiasis and assess its ability to enhance protective effects against *Schistosoma japonicum* infection. The PAMAM-Lys dendrimer based on PAMAM G4, which was modified with lysine, was synthesized (Figure 6).

Using PAMAM-Lys, complexes with a DNA vaccine against *Schistosoma japonicum* encoding the SjC23 membrane protein gene were obtained and characterized. To determine the most appropriate charge ratio (R+/2) of plasmid DNA and PAMAM-Lys/plasmid, complexes with various R+/2 ratios ranging from 0.5 to 10 were prepared, and the samples were analyzed by 1% agarose gel electrophoresis. The results showed that plasmid DNA exhibited complete retardation at a charge ratio of two with PAMAM G4 or four with PAMAM-Lys. Electron microscopy showed that the PAMAM-Lys/DNA complex forms particles with a diameter of 50 to 100 nm. It is important to note that PAMAM-Lys showed significantly higher transfection efficiency in 293T cells compared to native PMAM; in addition, the cytotoxicity of PAMAM-Lys was lower compared to that of PAMAM G5 [146].

An immunogenicity study showed that antibody titers in mice immunized with the combined PAMAM-Lys-SjC23 DNA vaccine were significantly higher than those in mice immunized with the naked SjC23 DNA vaccine. PAMAM-Lys elicited a predominantly humoral IgG2a response and dramatically increased IL-2 and IFN-γ production [146].

Immunization of mice with the SjC23 DNA vaccine in combination with PAMAM-Lys resulted in a 45–50% decrease in the number of helminths and a 59–62% decrease in the number of liver eggs, which is significantly higher than the effectiveness of the vaccine without SjC23 DNA. A 50–60% improvement in protection in the mouse model, as measured according to the reduction in the number of adult worms and eggs in the liver with a single antigen vaccine, is considered very encouraging. Thus, the SjC23 DNA vaccine with PAMAM-Lys provided an acceptable level of protective efficacy in the mouse model. It has been demonstrated that PAMAM-Lys dendrimers can increase the immunoreactivity of the DNA vaccine and enhance the protective effect of the SjC23 DNA vaccine against *S. japonicum* infection. The authors suggest that this new vaccine delivery vector could be an efficient and safe delivery vector for *S. japonicum* vaccine research and could also be used to develop other vaccines [146].

### 3.5. Dendrimers for the Delivery of DNA Vaccines against Cancer

Oncology is an area where dendrimers are widely used. They are employed both to deliver plasmids for cancer gene therapy [151] and as drug or small interfering RNA (siRNA) carriers, and can combine both payloads in a single preparation [23].

An original approach for delivery of antitumor DNA vaccines into anti-gen-presenting cells was suggested by Daftarian et al. [147]. This approach is based on the conjugation of fifth-generation polyamidoamine dendrimers (G5-PAMAM) with MHC class II–targeting peptides that can selectively deliver these dendrimers to APCs (Figure 6) [147].

The best results were obtained with the PADRE peptide conjugated to PAMAM. This peptide has a high affinity for more than 95% of all dietary leukocyte-D (HLA-DR) and murine H2-I-A^b^ antigens. PADRE-conjugated PAMAM dendrimer (PPD) was used to deliver the DNA vaccine pcDNA3-TRP2, encoding tyrosine-related protein-2, a melanocyte self-differentiation antigen. TRP2 is the main antigenic target of the immune response induced in mice by immunization with genetically modified B16 melanoma vaccines [147].

A comparative experiment was conducted in which C57BL/6 mice were immunized with pcDNA3-TRP2 or pcDNA3 using PPD or non-conjugated dendrimers. The immunization was performed using subcutaneous electroporation [147]. 

Daftaryan et al. showed that conjugation of PADRE peptide to dendrimers enhances the immune response obtained by the dendrimer, generating high affinity memory T cells capable of recognizing not only TRP2-pulsed MBL2 but also the rare MHC class I molecules endogenously loaded with TRP2 in the B16 melanoma [147].

Subcutaneous injection of DNA-peptide-dendrimer complexes in vivo preferentially transfected dendritic cells (DCs) in draining lymph nodes, promoted generation of highly effective T cells and induced rejection of established tumors. The delivery of pcDNA3-TRP2 via PPD leads to B16 tumor regression, and mice survival is roughly 50%. The results demonstrated that PADRE-PAMAM dendrimer complexes with DNA vaccines can be used for high transfection efficiency and effective targeting of APCs in vivo, conferring properties essential to generate effective DNA vaccines [147].

### 3.6. Dendrimers for the Delivery of RNA Vaccines for Treating Protein Metabolism Disorders

Messenger RNA can also be used as a platform for treating protein metabolism disorders.

Cheng et al. [148] studied the possibility of delivering mRNA encoding the fumarylacetoacetate hydrolase (FAH) protein gene using dendrimeric lipid nanoparticles (mDLNPs). In order to improve the therapeutic delivery of FAH mRNA to the liver, the authors obtained non-toxic dendrimeric lipid nanoparticles (mDLNPs) that should be well tolerated by mice with impaired liver function, as well as to ensure efficient packaging of long mRNAs.

**Figure 9:**
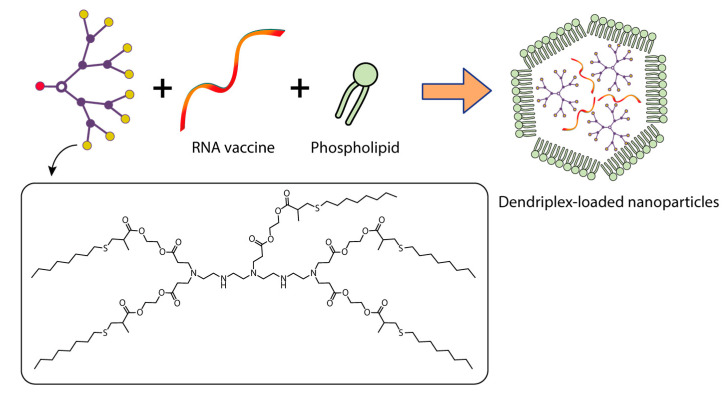
Constructing vaccine nanoformulations based on amphiphilic dendron 5A2-SC8.

They used a systematic orthogonal array design methodology designed to elucidate the functional contribution of each component of mDLNP nanoparticles for efficient mRNA delivery. 5A2-SC8 was chosen as an ionizable cationic dendrimer from a large library (Figure 9) because it had been successfully used previously by the authors to deliver siRNA to the liver to study gene functionality and showed very low toxicity. The incorporation of ionizable cationic lipids was necessary for RNA delivery because they bind RNA at low pH during mixing and promote intracellular release at lower pH during endosomal maturation. The phospholipid DOPE, which enhances mRNA loading and can form unstable hexagonal phases contributing to LDL disassembly and destabilization of the endosome membrane, was chosen [148].

In order to understand at what ratio each component of the nanoparticle composition should be taken, Cheng et al. used several rounds of optimization with testing of 44 mDLNP, which cover the theoretical space of 500 formulations. They found that LNPs optimized for mRNA delivery should contain significantly less ionizable cationic lipid and more zwitterionic phospholipids compared to standard siRNA formulations. Ultimately, the systematic optimization process allowed the development of a non-toxic, degradable delivery system that improved mRNA delivery to liver hepatocytes. mDLNPs were monodisperse particles with a diameter of ~100 nm and an almost neutral surface charge (−3.58 mV). Due to the high transfection efficiency in vivo, mDLNP 5A2-SC8 was able to extend the lives of FAH−/− knockout mice. The FAH mRNA treatment normalized body weight and liver function throughout the 30-day experiment. The authors believe that the ability of mDLNP 5A2-SC8 to deliver FAH mRNA to the diseased liver without carrier toxicity makes this system suitable for treating a wide range of liver diseases [148].

### 3.7. The Use of Complexes of Dendrimers with Metal Nanoparticles for mRNA Delivery

Recently, several studies have used the remarkable properties of dendrimers as stabilizers of metal nanoparticles (NPs) [152,153,154,155,156,157]. This strategy combines the unique properties of metal NPs with those of cationic dendrimers to create safe and highly efficient non-viral gene delivery systems. Gold nanoparticles (AuNPs) are among the most commonly used metal NPs today due to their ease of synthesis, biocompatibility, favorable surface-to-volume ratio, ability to be modified, and low cytotoxicity [158,159].

**Figure 10:**
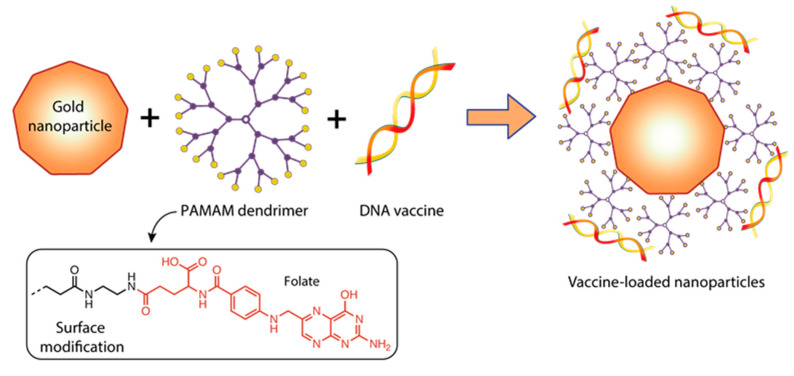
Constructing vaccine nanoformulations based on dendrimer-functionalized gold nanoparticles.

Mbatha et al. [149] studied the original complexes of PAMAM G5D dendrimers with gold nanoparticles (AuNPs). The objective of their research was to generate folic-acid-(FA)-modified, poly-amidoamine-generation-5 (PAMAM G5D)-grafted gold nanoparticles (AuNPs) and evaluate their cytotoxicity and efficacy profiles for in vitro mRNA delivery (Figure 10).

The mRNA encoding the luciferase gene (FLuc-mRNA) was used as a model. Mbatha et al. obtained a number of nanocomplexes that contained a constant amount of FLuc-mRNA (0.05 µg) together with increasing amounts of G5D, Au:G5D, G5D:FA, and Au:G5D:FA NPs. The NPs appeared spherical with a uniform distribution and hydrodynamic diameters ranging from 65 to 128 nm. Nanocomplexes with mRNA prepared at the optimum binding ratios (*w*/*w*) are presented as clusters of smaller particles with hydrodynamic diameters ranging from 101 nm to 265 nm. The zeta potentials generally ranged from 20.9 to 87.2 mV for the NPs and from −21.0 to −65 mV for the nanocomplexes, indicating good colloidal stability. Au:G5D and Au:G5D:FA nanocomplexes had the highest zeta potentials of −37.3 mV and −65.7 mV, respectively. The polydispersity indices (PDI) revealed that all the NPs and nanocomplexes were highly monodisperse and uniform in size, with PDI values below 0.2, suggesting that these NPs and nanocomplexes have a lower tendency to agglomerate [149].

The authors demonstrated that nanocomplexes at optimum nanoparticle:mRNA (*w*/*w*) binding ratios showed good protection of the bound mRNA against nucleases and were well tolerated in all tested cell lines. The transfection efficiency and luciferase gene expression levels were significantly higher with FA-targeted, dendrimer-grafted AuNPs (Au:G5D:FA) in FA receptors overexpressing MCF-7 and KB cells compared to the G5D and G5D:FA NPs, decreasing significantly in the presence of excess competing FA ligand, which confirmed the nanocomplex uptake via receptor mediation [101]. The level of Luc gene expression was higher when Au:G5D and Au:G5D:FA nanocomplexes with mRNA were used to transfect cells compared to when G5D and G5D:FA nanocomplexes were employed; this fact indicates the important role played by AuNP in this delivery system [149].

## 4. Discussion

**Figure 11:**
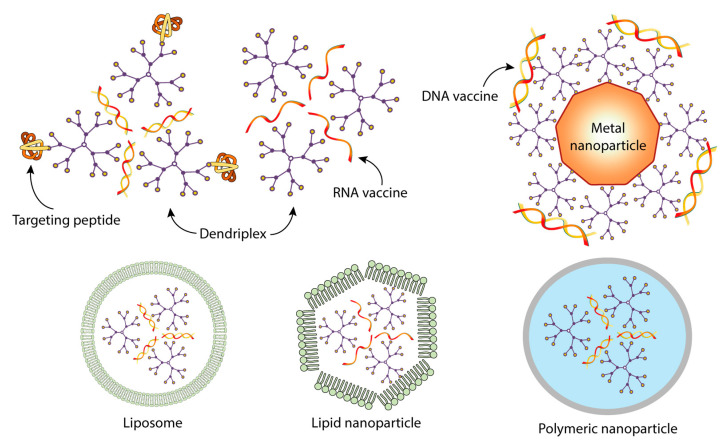
Typical structures of dendrimer-based complexes used for the delivery of DNA and RNA vaccines.

In the studies presented in Table 2, the immunogenicity of nanoformulations of vaccine constructs and dendrimers (Figure 11) was significantly higher than when using a naked DNA or mRNA vaccine. 

In all cases, cationic dendrimers were used to prepare vaccine nanoformulations. Cationic dendrimers, due to the presence of a large number of positively charged groups, are able to effectively bind and condense nucleic acids (NA) into more compact forms. The electrostatic interactions are therefore the main driving force of the formation of nanosized polyelectrolyte complexes. Complexes bearing a cationic surface charge bind more efficiently to cell membranes and then penetrate into cells via endocytosis, while other mechanisms, including the receptor-dependent pathway, are possible [160]. Alternatively, dendrimer-NA complexes are entrapped into lipid-based nanoparticles, with both physical entrapment and van der Waals interactions between components involved in this process. Whereas the use of natural lipids favors the formation of vesicles (liposomes), the rational design of synthetic lipids may drive the association of complexes and lipids into hexagonal-phase lipid nanoparticles, depending on the molecular topology of a lipid. 

Among the different types of cationic dendrimers for the delivery of DNA vaccines, the leaders are polyamidoamine (PAMAM) dendrimers generations one through five from the first to the fifth generation (G1, G2, G4, or G5) [139,140,141,142,143,146,147,149] (Figure 6). Dendrimers of small generations have an open, mobile, and asymmetric structure, and with increasing generation this structure becomes more globular and densely packed. The rigidity of PAMAM dendrimers and the efficiency of their binding to the nucleic acid increase with the increasing number of generations; however, the toxicity of dendrimers also increases. Therefore, it is necessary to find a balance between delivery efficiency and toxicity. In recent years, researchers have preferred to use PAMAM G4 [139] or G5 [149] for DNA vaccine delivery. It should be noted that PAMAM dendrimers have shown their safety in clinical trials at Ashvattha Therapeutics [131].

If a target molecule is covalently attached to the amino groups of PAMAM, then the complex can be delivered to target cells in this way. In the development of cancer vaccines, researchers have used PAMAM conjugated with MHC and PADRE epitope molecules as an efficient transporter of the DNA vaccine to the cells of the immune system [147]. 

Interestingly, the PAMAM G5 complex with modified folic acid gold nanoparticles proved to be useful for mRNA delivery [149]. Using this complex, the authors managed to significantly increase the efficiency of transfection of eukaryotic cells with mRNA [149]. The nanoparticle:mRNA complex (*w*/*w*) showed good protection of the bound mRNA from nucleases, and folic acid allowed the complex to effectively enter MCF-7 cells through the corresponding receptors.

In earlier works, polypropylene imine (PPI) dendrimers, which have a 1,4-diaminobutane core and propylene imine monomer groups, were used to deliver DNA vaccines [138]. The use of the PPI G5—DNA vaccine for the immunization of animals caused a slight increase in the immune response. The attachment of phosphatidylcholine and cholesterol to PPI, which increased the tropism of the complex to membranes, significantly increased the immunogenicity of the dendrosome composition [138].

A fourth-generation amine-terminated poly(ether imine) dendrimer (PETIM) was tested as a DNA vaccine carrier, which increased immunogenicity and delivery efficiency [137]. However, no more work has been published using the PETIM dendrimer in the last five years.

Attempts have been made to use polylactide-co-glycolide (PLGA) particles to deliver a DNA vaccine encoding a *Bacillus anthracis* antigen. PLGA-C(G_18_) dendriplex particles were more immunogenic than PLGA-C(G_0_) and induced specific antibodies; however, these antibodies were not able to neutralize the *Bacillus anthracis* toxin [144]. 

Thus, the data presented in Table 2 demonstrate the usefulness of cationic dendrimers, primarily PAMAM dendrimers, for use in research as a method for delivering DNA and mRNA vaccines against viral [137,138,139,140,141,142,143], bacterial [144,145], parasitic infections [146], and oncological diseases [147]. Most of the work was devoted to the delivery of DNA vaccines, and so far, only a few works have been devoted to the delivery of mRNA, since this is still a fairly new direction in vaccinology.

## 5. Conclusions and Perspectives

In this review, we set out to demonstrate the growing interest in dendrimers as carriers of DNA and mRNA vaccines, which can become an alternative to other delivery methods. Currently, vaccines based on viral vectors [161,162] and self-replicating RNA [163] can be named among the most effective methods for delivering nucleic acids. The disadvantage of viral vectors is their reactogenicity, safety limitations, high production costs, and the problem of the immunogenicity of the vectors themselves. As a result, anti-vector immune response reduces the effectiveness of booster immunizations with the same virus. Jet injection was used to deliver a DNA vaccine against COVID-19 developed by Zydus Cadila. However, to achieve high efficacy, the patient must be administered a sufficiently high dose of plasmid DNA (2 mg/dose).

At present, lipid nanoparticles are the leading construction in nucleic acid delivery. They are a vital component of the new Pfizer/BioNTech and Moderna mRNA COVID-19 vaccines, playing a key role in protecting and transporting the mRNA effectively to the right place in cells [22]. The most important advantages of lipid nanoparticles are the ease of scaling up production, the biocompatibility and biodegradability of the formulation components, and the possibility to obtain them loaded both with hydrophilic and lipophilic therapeutic agents. However, the delivery of mRNA by lipid nanoparticles incorporating cationic lipids has a number of obstacles related to the nature of lipids. In particular, cationic liposomes can attach to the cell surface, thereby contributing to the destabilization of the plasma membrane and causing side effects in a vaccinated person [23,24]. They can cause an allergic reaction. In addition, cases of reactions characterized by pericarditis, myocarditis, facial paralysis, and other symptoms have been reported in patients vaccinated with COVID-19 mRNA vaccines [23,24]. Furthermore, as mentioned above, liposome-based vaccine formulations require special production, transportation, and handling conditions.

Dendrimers as delivery systems have a number of useful properties, such as monodispersity, biocompatibility, low reactogenicity, high biodegradability, water solubility, simplicity, and a relatively low cost of synthesis [84,164,165,166]. Dendrimers often exhibit significant adjuvant effects in vaccine delivery because they can be readily taken up by antigen-presenting cells. In addition, cationic dendrimers form more stable complexes with NA, providing greater protection during cellular transport than cationic lipids [167].

Despite all the positive properties of dendrimers, until recently, the possibility of clinical use was constrained by their relative toxicity. There are several reasons behind the toxicity associated with dendrimers. One of the reasons is their high cationic charge and the presence of amino groups, which can damage the cell membrane, including erythrocytes. Meanwhile, anionic dendrimers containing dCOOH and dOH groups are less toxic than cationic ones. A study of PAMAM dendrimers with terminal amino groups (G3.0, G5.0, and G7.0) using laboratory animals showed that their toxicity increased with the number of amine generations and cationic charge and also depended on the dose of the administered dendrimer [168]. There are many articles describing the cationic charge of dendrimers in terms of their toxicity with respect to biological membranes [160,169,170].

Currently, work is underway to improve dendrimer safety. For instance, surface modification is widely used for reducing dendrimer toxicity. Often used for this purpose is the grafting of biomimetic molecules such as PEG (polyethylene glycol) or biomacromolecules (peptides, antibodies, proteins, etc.). These molecules are involved both in masking the toxicity of the complex and in targeting the dendrimer to the appropriate target, which improves complex uptake efficiency, pharmacokinetics, and transfection efficiency [171,172,173,174,175]. Among other approaches, noteworthy is the acetylation of dendrimers, which leads to neutralization of the surface amine cationic charge, which causes dendrimer toxicity by disrupting biological membranes. It is important to note that the very process of dendrimer complexation with nucleic acids (including short oligonucleotides, miRNAs, RNA, and DNA vaccines) reduces the charge of the complex and the cytotoxicity of the dendrimer [176,177,178]. Thus, dendrimer toxicity issues can be successfully addressed using chemical and biophysical approaches.

Distinct dendrimer-based constructs have been clinically tested and have been shown to be safe and effective in Phases 1–3, with some studies still ongoing. Table 1 lists dendrimer-based products in clinical trials, according to the Clinictrials.gov database. These examples clearly highlight the growing use of dendrimer-based drug delivery systems in the pharmaceutical industry.

It is also clear that the potential of dendrimers as carriers of nucleic acid-based vaccines is far from being fully explored. By varying the structure of the terminal groups of the dendrimer, one can control the charge, hydrophobicity, and, ultimately, the pharmacokinetics of vaccine-carrier complexes. It should be emphasized that in order to increase the efficiency of NA delivery into antigen-presenting cells, dendrimer-based vaccine formulations with DNA or RNA may include other components such as targeting peptide moieties. Dendriplexes can also be packaged into easily internalizable nanoparticles, such as liposomes, polymer nanoparticles, etc. The complexation can not only protect NA vaccine constructions from digestion, but also provides targeted transport of NA to specific host cells, for example, cells of the immune system, mucous membrane, etc. The delivery targeting can be increased by introducing targeting moieties or ligands in the dendrimer structure. Being monodisperse polymers, dendrimers have a specific immunomodulatory activity, but the use of dendrimers as independent adjuvants has not yet been studied enough.

Thus, dendrimers represent a useful platform for the development of safe vaccines with new properties and application potential and will also be useful for basic research on the mechanisms underlying the induction and control of immunity.

## Figures and Tables

**Table 1:** Dendrimer-based drugs in clinical trials.

Drug	Description of the Drug	Study Title	Study Dates	Brief Study Description	Company	Clinicaltrials.gov Identifier	Ref.
SPL-7013 Gel (VivaGel™)	G4 poly(L-lysine) dendrimer bearing 32 sodium 1-(carboxymethoxy) naphthalene 3,6-disulfonate on the surface	SPL7013 gel—male tolerance study	August 2006–June 2007	A phase 1, placebo-controlled study of the safety of a 3% *w*/*w* SPL7013 gel, administered to the penis of healthy male volunteers once daily for seven days	Starpharma Pty Ltd., Abbotsford, Australia	NCT00370357	[87]
SPL-7013 Gel (VivaGel™)	“	VivaGel™ in healthy young women	December 2006–November 2007	A phase 1, expanded, randomized placebo-controlled trial of the safety and tolerability of a 3% *w*/*w* SPL7013 gel in healthy young women when administered twice daily for 14 days	Starpharma Pty Ltd., Abbotsford, Australia	NCT00331032	[88]
SPL-7013 Gel (VivaGel™)	“	Safety and acceptability of SPL7013 gel (VivaGel™) in sexually active women	July 2007–December 2009	A phase 1 study of the safety and acceptability of a 3% *w*/*w* SPL7013 Gel applied vaginally in sexually active young women	Starpharma Pty Ltd., Abbotsford, Australia	NCT00442910	[89]
SPL-7013 Gel (VivaGel™)	“	Retention and duration of activity of SPL7013 (VivaGel^®^) after vaginal dosing	August 2008–March 2009	Phase 1 and phase 2 assessments of local retention and duration of activity following vaginal application of a 3% VivaGel in healthy volunteers	Starpharma Pty Ltd., Abbotsford, Australia	NCT00740584	[90]
SPL-7013 Gel (VivaGel™)	“	Dose-ranging study of SPL7013 gel for treatment of bacterial vaginosis (BV)	August 2010–May 2011	A phase 2, double-blind, multicenter, randomized, placebo-controlled, dose-ranging study to determine the efficacy and safety of the VivaGel administered vaginally in the treatment of bacterial vaginosis	Starpharma Pty Ltd., Abbotsford, Australia	NCT01201057	[91]
SPL-7013 Gel (VivaGel™)	“	Dose-ranging study of SPL7013 gel for the prevention of bacterial vaginosis (BV)	August 2011–December 2012	A phase 2, double-blind, multicenter, randomized, placebo-controlled, dose-ranging study to determine the efficacy and safety of the SPL7013 gel administered vaginally to prevent the recurrence of bacterial vaginosis	Starpharma Pty Ltd., Abbotsford, Australia	NCT01437722	[92]
SPL-7013 Gel (VivaGel™)	“	A phase 3 study of SPL7013 gel (VivaGel) for the treatment of bacterial vaginosis	April 2012–October 2012	A phase 3, double-blind, multicenter, randomized, placebo-controlled study to assess the efficacy and safety of a 1% SPL7013 gel for the treatment of bacterial vaginosis	Starpharma Pty Ltd., Abbotsford, Australia	NCT01577537	[93]
SPL-7013 Gel (VivaGel™)	“	A phase 3 study of SPL7013 gel (VivaGel) for the treatment of bacterial vaginosis	March 2012–July 2012	A phase 3, double-blind, multicenter, randomized, placebo-controlled study to assess the efficacy and safety of a 1% SPL7013 gel for the treatment of bacterial vaginosis	Starpharma Pty Ltd., Abbotsford, Australia	NCT01577238	[93]
SPL-7013 Gel (VivaGel™)	“	Efficacy and safety study of SPL7013 gel to prevent the recurrence of bacterial vaginosis (BV)	October 2014–October 2016	A phase 3, double-blind, multicenter, randomized, placebo-controlled study to determine the efficacy and safety of the SPL7013 gel to prevent the recurrence of bacterial vaginosis	Starpharma Pty Ltd., Abbotsford, Australia	NCT02236156	[94]
SPL-7013 Gel (VivaGel™)	“	Efficacy and safety study of SPL7013 gel to prevent the recurrence of bacterial vaginosis (BV)	October 2014–February 2017	A phase 3, double-blind, multicenter, randomized, placebo-controlled study to determine the efficacy and safety of the SPL7013 gel to prevent the recurrence of bacterial vaginosis	Starpharma Pty Ltd., Abbotsford, Australia	NCT02237950	[95]
AZD0466	Astra Zeneca cancer drug AZD4320, chemically conjugated to a PEGylated poly-lysine dendrimer	A Study of AZD0466 in patients with advanced hematologic or solid tumors	December 2019–June 2021	A phase 1, first-in-human study to determine the safety, tolerability, maximum tolerated dose (MTD), recommended Phase 2 dose (RP2D), and pharmacokinetics (PK) of AZD0466 in patients with solid tumors, lymphoma, and multiple myeloma at low, intermediate, or high risk for tumor lysis syndrome (TLS) with hematologic malignancies for whom no standard therapy exists	Starpharma, Abbotsford, Australia; AstraZeneca, UK, Cambridge	NCT04214093	[96]
AZD0466	“	A phase I/II study of AZD0466 as monotherapy or in combination with anticancer agents in advanced non-Hodgkin lymphoma	July 2022–November 2024 [Estimated]	A phase 1/2, modular, open-label, dose escalation and expansion, multicenter study of the safety, tolerability, PK, and preliminary efficacy of AZD0466 as a monotherapy, or in combination with other anticancer agents in patients with advanced NHL	Starpharma, Abbotsford, Australia; AstraZeneca, UK, Cambridge	NCT05205161	[97,98]
AZD0466	“	Study of AZD0466 monotherapy or in combination in patients with advanced hematological malignancies	June 2021–June 2024 [Estimated]	A phase 1/2, modular, open-label, multicenter study to assess the safety, tolerability, pharmacokinetics, and preliminary efficacy of AZD0466 as a monotherapy and drug-drug interaction potential between AZD0466 and the azole antifungal voriconazole in participants with advanced hematological malignancies	Starpharma, Abbotsford, Australia; AstraZeneca, UK, Cam-bridge	NCT04865419	[99]
ImDendrim	G5 polylysine dendrimer mixed with nitro-imidazole-methyl-1,2,3-triazol-methyl-di-(2-pycolyl) amine	Treatment of non-responding to conventional therapy inoperable liver cancers by in situ introduction of ImDendrim	March 2017–December 2017	An open-label and unicenter study in patients with primary hepatocellular cancer or metastatic liver cancer without standard therapeutic options for treatment, including chemotherapy or surgery	National Institute of Allergy and Infectious Diseases (NIAID), North Bethesda, Maryland, USA	NCT03255343	[100]
OP-101	G4 PAMAM dendrimer N-acetyl-cysteine	A study to evaluate the safety, tolerability, and pharmacokinetics of OP-101 after intravenous administration in healthy volunteers	March 2018–July 2018	A phase 1, open-label single ascending dose study to evaluate the safety, tolerability, and pharmacokinetics after intravenous administration in healthy volunteers	Orpheris, Inc. Redwood City, California, USA	NCT03500627	[101]
OP-101	“	A clinical study to measure the effect of OP-101 after being administered subcutaneous in healthy volunteers	March 2020–May 2020	A phase 1, open-label single ascending dose study to evaluate the safety, tolerability, and pharmacokinetics after subcutaneous administration in healthy volunteers	Orpheris, Inc. Redwood City, California, USA	NCT04321980	[102]
OP-101	“	A study to evaluate OP-101 (dendrimer N-acetyl-cysteine) in severe coronavirus disease 2019 (COVID-19) patients (PRANA)	August 2020–August 2022 [Estimated]	A phase 2, two-stage, double-blind, placebo-controlled study to evaluate the safety, tolerability, pharmacokinetics, and efficacy in patients with severe COVID-19	Ashvattha Therapeutics, Inc. Redwood City, California, USA	NCT04458298	[103]
D-4517.2	Hydroxyl dendrimer, VEGFR tyrosine kinase inhibitor	A study to evaluate the safety, tolerability, and pharmacokinetics of D-4517.2 after subcutaneous administration in healthy participants	January 2022–August 2022	A phase 1, open-label, single-ascending dose study of the safety, tolerability, and pharmacokinetics after subcutaneous administration in healthy volunteers	Ashvattha Therapeutics, Inc. Redwood City, California, USA	NCT05105607	[104]
D-4517.2	“	A study to evaluate the safety, tolerability, and pharmacokinetics of D-4517.2 after subcutaneous administration in subjects with neovascular (Wet) age-related macular degeneration (AMD), or subjects with diabetic macular edema (DME) (Tejas)	August 2022–June 2023 [Estimated]	A phase 2, two-stage study: open-label assessment of safety and pharmacodynamic response as well as a visual examiner-masked, randomized active, sham, and placebo controlled study evaluating the efficacy of D-4517.2 administered subcutaneously to subjects with neovascular age-related macular degeneration or subjects with diabetic macular edema	Ashvattha Therapeutics, Inc. Redwood City, California, USA	NCT05387837	[105]
siCoV/KK46	Anti-SARS-CoV-2 siRNA (targeting RNA-dependent RNA polymerase)/KK-46 (peptide dendrimer) complex	The siCoV/KK46 drug open-safety study	January 2021–March 2021	A phase 1, open-label, dose-escalation study to assess the safety and tolerability of single and multiple doses in healthy volunteers (inhalation use)	National Research Center —Institute of Immunology FMBA, Saint Petersburg, Russia	NCT05208996	[106]
MIR 19^®^	“	Evaluation of safety and efficacy of a MIR 19 ^®^ inhalation solution in patients with moderate COVID-19	April 2021–September 2021	A phase 2, multicenter controlled randomized study to assess the efficacy and safety of MIR 19^®^ via 14 days of treatment of participants with symptomatic moderate COVID-19	National Research Center—Institute of Immunology FMBA, Saint Petersburg, Russia	NCT05184127	[107]

**Table 2:** Dendrimers for the delivery of DNA and mRNA vaccines.

Vaccine Antigen/Type of Tumor	Complex Type	Physicochemical Characteristics of Particles	Immunization			Immune Response	Ref.
			Model	Administration Route/Regimen	Dose		
Viral infections							
DNA vaccine							
Rabies surface glycoprotein (Rgp)	Dendriplex PETIM:pIRES-Rgp	PETIM:pIRES-Rgp ratio (*w/w*)—10:1; Particle size—500 nm	Swiss albino mice	IM/triple injection on days 0, 7, and 21	90 μg of pIRES-Rgp 10 μg of PETIM: pIRES-Rgp	Immunization PETIM:pIRES-Rgp provided induction of specific anti-rabies IgG starting from the 14th day and provided 100% protection of animals against virus infection	[137]
Hepatitis B surface antigen	Dendriplex: pRc/CMV-HBs/ Poly(propylene imine) dendrimer PPI G5 DF3: Dendriplex-loaded phosphatidylcholine (PC) and cholesterol (C) vesicles	Dendriplex PPI 50: Molar ratio plasmid: PPI—1:50 Zeta potential (mV)—21.3 ± 0.33 DF3: Molar ratio PC:C—7:3 PPI 50 entrapment efficiency (%)—46.79 ± 1.33 Vesicle size (nm)—121 ± 2.9 Zeta potential (mV)—29.33 ± 0.21	Balb/c mice	IM/single injection on day 1	10 µg of plasmid pRc/CMV-HBs in dendrimer or dendrosome form	Complex DF3 provided the induction of specific anti-HBs IgG and Th1 response significantly higher and longer than complex PPI 50. The “naked” pRc/CMV-HBs had weak immunogenicity.	[138]
Ebola virus	Dendriplex PAMAM G4 dendrimer + DNA encoding artificial T-cell antigens EBOV EV.CTL and EV.Th	pEV.CTL/pEV.Th + PAMAM N/P ratio—3:1; Particle size (nm)—< 100; Zeta Potential, (mV): pEV.CTL + PAMAM—27.3 ± 6.9; pEV.Th + PAMAM—9.6 ±6.7	Balb/c mice	IM/triple injection on days 0, 14, and 28	100 μg of plasmid pEV.CTL /pEV.Th + PAMAM G4	The immune response to both naked DNA vaccines and DNA vaccines in combination with PAMAM was the same.	[139]
H5N1 avian influenza virus	Dendriplex PAMAM G5 dendrimer + TAT polypeptide + DNA (pBud-H5-GFP)	TAT-PAMAM-DNA polyplexes: Molar ratio—6:1 Average particle size (nm)—105 Zeta potential (mV)—42	Balb/c mice	IM/ double injection on days 0 and 21	50 μL of PAMAM—pDNA polyplexes and TAT-PAMAM-pDNA polyplexes	Immunization with TAT-PAMAM-DNA and PAMAM-DNA polyplexes caused the formation of specific HI-antibodies and induced T-cell activation	[140,141]
		PAMAM—DNA polyplexes: Molar ratio—6:1 Average particle size (nm)—103 Zeta potential (mV)—32					
mRNA-vaccine							
(a) Ebola virus glycoprotein (b) H1N1 influenza hemagglutinin (c) *Toxoplasma gondii*	Amphiphilic dendrimer + replicating VEEV mRNAs encoding pathogen antigens	Mass ratio of modified dendrimer to 1,2-dimyristoyl-sn-glycero-3-phosphoethanolamine-N-[methoxy(polyethylene glycol)-2000] to RNA—11.5:1:2.3 Diameter (nm)~ 200–300	C57BL/6 mice	IM/single injection on day 1	40 µg of MDNP-encapsulated VEEV RNAs encoding different pathogen antigens	Immunization with PAMAM G1-mRNAs caused the formation of IgG and induced T-cell activation. Immunization of PAMAM G1-mRNA encoding the Ebola virus antigens provided 60% protection of animals from virus infection. Immunization of PAMAM G1-mRNAs encoding H1N1 and *Toxoplasma gondii* antigens ensured 100% protection of animals against virus infection.	[142]
Zika virus	Amphiphilic dendrimer + RNA, VEEV with RNA encoding ZIKV E protein	Mass ratio of modified dendrimer to 1,2-dimyristoyl-sn-glycero-3-phosphoethanolamine-N-[methoxy(polyethylene glycol)-2000] to RNA—11.5:1:2.3 Diameter (nm)~ 200–300	Balb/c mice	IM/single injection on Day 1	40 µg of MDNP-encapsulated VEEV RNAs, encoding ZIKV E protein	Immunization with PAMAM G1-mRNA caused the formation of IgG and induced T-cell activation.	[143]
Bacterial infections							
Protective antigen (PA) gene of *Bacillus anthracis*	Amphiphilic poly-L-lysine dendron + PA antigen DNA encapsulated in PLGA particles	PLGA-PA DNA: with the same molar charge ratio Mean particle size (nm)—> 800 Zeta Potential, (mV)~ −19	Balb/c mice	IM/quadruple injection on days 1, 21, 42, and 63	Prime injection: 20 µg of PLGA-PA DNA or DNA:Dendron Boost injection: 14 µg of PLGA-PA DNA or DNA:Dendron	Immunization with PLGA-PA DNA induced specific IgG1 but was not able to neutralize the toxin	[144]
		DNA:Dendron: Molar charge ratio—10:1; Mean particle size (nm)~ 400; Zeta Potential, (mV)~ −17					
*Chlamydophila (Cp.) psittaci*	Poliplex brPEI-pcDNA1/MOMP_opt_	brPEI polyplexes: *N/P ratio—8;* *Particle size (nm)~ 114;Zeta Potential, mV~ 48*	Turkeys	IM / aerosol/ double injection on days 1 and 21	100 µg of plasmid pcDNA1/MOMP_opt_ and brPEI-pcDNA1/MOMP _opt_ (IM);	Immunization with brPEI polyplex induces the formation of specific IgG, identifying a significantly higher average percentage of CD4^+^ T cells and provides a high level of protection against virus infection for animals	[145]
					500 µg of brPEI-pcDNA1/MOMP _opt_ (Aerosol)		
Parasitic infections							
*Schistosoma japonicum*	Dendriplex G4 PAMAM-Lys + membrane protein DNA (SjC23)	PAMAM-Lys/DNA complex Charge ratio—4:1; Particle size (nm)–50–100;	Balb/c mice	IM/ Triple injection on days 0, 14, and 28	100 μg of plasmid PAMAM-Lys/DNA complex	Immunization with PAMAM-Lys elicited a predominantly humoral IgG2a response and a dramatic increase in IL-2 and IFN-γ production compared to the SjC23 naked DNA vaccine.	[146]
Oncological diseases							
Melanoma	DNA (pcDNA3-tyrosine-related protein-2 (TRP2) and pcDNA3-gp70) conjugated with G5-PAMAM-PADRE epitope	N/P ratio—10:1; Particle size (nm)–600;	mice C57BL/6	Subcutaneous electroporation/ double injection on days 0 and 14	20 μg of plasmid pcDNA3-TRP2	Subcutaneous injection of DNA-peptide-dendrimer complexes, followed by dermal electroporation, transfected APC, mostly DCs, in vivo directly in the lymph nodes, induced T cell immunity and humoral response, and reduced tumor growth in a B16F10 melanoma model.	[147]
Protein metabolism disorders							
mRNA-FAH (fumarylacetoacetate hydrolase)	5A2-SC8-mRNA-loaded dendrimer lipid nanoparticles (mDLNPs)	5A2-SC8 + mRNA Mass ratio—20:1; Diameter (nm)—95–101; Zeta Potential, (mV)—3.58	Balb/c mice	IM/single injection on Day 1	0.5 µg of mDLNPs	mDLNPs transfect >44% of all hepatocytes in the liver and produce high levels of FAH protein	[148]
The use of complexes of dendrimers with metal particles for mRNA delivery							
FLuc-mRNA	Gold nanoparticles modified with folate-conjugated PAMAM G5 complexed with FLuc-mRNA	Au:G5D:FA-mRNA: NP:mRNA(*w*/*w*) Ratio–4:1 Mean Diameter(nm) ± SD— 101.8 ± 36.9 Zeta Potential (mV) ± SD—65.7 ± 1.4 Polydispersity Index—0.131	Cell lines: HEK293, HepG2, MCF-7, KB and Caco-2	-	0.05 µg of Au:G5D:FA+ FLuc-mRNA	Folic acid modification of Au:G5D:FA + FLuc-mRNA nanoparticles with grafted gold particles resulted in higher transfection efficiency in all cell lines. The use of G5 dendrimer increased stability of mRNA molecule.	[149]

Note: PETIM—poly (ether) imine dendrimer; PPI—polypropyleneimine dendrimer; G—generation; DF3—dendrosomal formulication—3; PAMAM—polyamidoamine dendrimer; brPEI—branching polyethyleneimine; IRES—internal ribosome entry site; CMV—*Cytomegalovirus;* IM—intramuscularly; NP—ratio of nitrogen to phosphorus; mDLNPs—mRNA-loaded dendrimer lipid nanoparticles; PLGA—poly(lactide-co-glycolide) particles; FAH—fumarylacetoacetate hydrolase; FA—folic acid; PC—phosphatidylcholine; C—cholesterol; TRP2—tyrosine related protein 2; VEEV—Venezuelan equine encephalitis virus; DCs—dendritic cells; APC—antigen presenting cells; FLuc-mRNA—mRNA encoding the luciferase gene; SjC23—membrane protein of *Schistosoma japonicum;* pDNA/MOMP_opt_—plasmid containing optimized sequences of the transgene and regulatory elements; TAT—transactivator of transcription protein of HIV-1; Il-2—interleukin 2; IFN-γ—interferon gamma; HEK293, HepG2, MCF-7, KB and Caco-2—cell lines.

## Data Availability

Not applicable.
